# Properties of n-Octadecane PCM Composite with Recycled Aluminum as a Thermal Enhancer

**DOI:** 10.3390/ma18245638

**Published:** 2025-12-15

**Authors:** Nicoleta Cobîrzan, Gyorgy Thalmaier, Crețu Mihaela, Mircea Năsui, Dan Doru Micu

**Affiliations:** 1 Faculty of Civil Engineering, Buildings and Management Department, Technical University of Cluj-Napoca, 15 Constantin Daicoviciu Street, 400020 Cluj-Napoca, Romania; nicoleta.cobarzan@ccm.utcluj.ro; 2 Institute of Nanomaterials and Nanotechnologies—EUTINN, European University of Technology, European Union, 28 Memorandumului Street, 400114 Cluj-Napoca, Romania; mihaela.cretu@ethm.utcluj.ro (C.M.); mircea.nasui@chem.utcluj.ro (M.N.); dan.micu@ethm.utcluj.ro (D.D.M.); 3 Faculty of Materials and Environmental Engineering, Material Science and Engineering Department, Technical University of Cluj-Napoca, 103 Muncii Blv., 400641 Cluj-Napoca, Romania; 4 Faculty of Electrical Engineering, Electrotechnics and Measurements Department, Technical University of Cluj-Napoca, 26, G. Baritiu Str., 400027 Cluj Napoca, Romania; 5 Faculty of Materials and Environmental Engineering, Physis and Chemistry Department, Technical University of Cluj-Napoca, 103 Muncii Blv., 400641 Cluj-Napoca, Romania

**Keywords:** recycled aluminum chips, PCM composite, thermal conductivity enhancement, phase-change kinetics, passive cooling

## Abstract

This paper presents new types of PCM composites proposed and analyzed for cooling applications in buildings. The composites (n-octadecane-Al-long/n-octadecane-Al-short) were made of n-octadecane with 7% and 7.5% vol. of recycled aluminum added as a thermal conductivity enhancer to avoid sinking during the melting phase and to improve thermal conductivity. Recycled aluminum chips are inexpensive, abundant, and generate a lower environmental impact during composite production. The effect of the chip content was found to increase the thermal conductivity values of the composites by 100% (n-octadecane-Al short) and by 600% (n-octadecane-Al-long) compared to n-octadecane. The percentage of mass increase remained low. The latent heat of n-octadecane-Al-long decreased from 245 kJ/kg to 195 kJ/kg, the melting time shortened from 990 s to 850 s, and the CO_2_ emission reduction was by 150 kg CO_2_eq/year. The volume of the PCM composites varied from 0.083 m^3^ (n-octadecane) to 0.091 for n-octadecane-Al-long, which represents an increase of up to 11% needed to absorb the solar heat gained by the optimized PCM composite.

## 1. Introduction

The rising level of environmental pollution has intensified the global warming effect in the past century and produced major climate transformations. The rise in extreme heat increases the cooling demands of buildings [1,2] and raises their maintenance costs. Cluj-Napoca, Romania, has a moderate continental climate, with temperatures higher than they were in past decades, which can now reach up to 40 °C during summer days. Inside buildings, especially those that have not been thermally rehabilitated, the indoor temperature can reach up to 30 °C, which is above thermal comfort conditions. In this regard, HVAC systems become essential for ensuring indoor comfort and the well-being of residents; however, they consume energy for air conditioning, generate greenhouse gas (GHG) emissions, and increase the operational costs of buildings. Phase change materials (PCMs) used in passive design have been found to be an effective alternative solution to overcome these effects by saving energy in buildings or in other sectors [3,4,5,6,7,8,9,10]. During melting/solidification [11], PCMs have the ability to store/release latent energy [12,13], thus passively balancing the indoor temperature to maintain thermal comfort.

Kalbasi & Hassani [14] numerically investigated the thermal behavior of buildings under ten climatic conditions, reporting an improvement in thermal comfort of up to 83% due to the presence of PCM. The influence of a PCM layer in the structure of an external wall was also analyzed by Alshuraiaan [15], who highlighted its significant influence on regulating the temperature of the indoor environment. Numerical calculations performed by Nizovtsev & Sterlyagov [16] show the positive effects of PCM in increasing the thermal inertia of lightweight buildings, being effective in reducing the amplitude of heat flux through walls and in saving energy. Al Jebaei et al. [12] emphasize the importance of selecting the PCM type for higher performance in saving HVAC energy and CO_2_ emissions. They reported that an optimal transition range and melting temperatures of PCM prove their effectiveness in the range of external climate conditions.

Organic PCMs have lower thermal conductivity, which seems to be a disadvantage for a material used as a thermal energy system [17,18]. To increase the thermal conductivity of PCMs and the rate of heat charging/discharging [19], additives such as metals, carbon, and other based materials [20,21,22,23,24] have been proposed by different authors, and their performance has been highlighted in accordance with their role and field of application.

Despite the maturity of PCM research, a systematic review reveals clear and consistent gaps. Most studies suggest two ways to enhance the thermal conductivity of PCM [25,26,27,28,29,30]. One is by impregnating the PCM in a high-porosity, high-conductivity metallic foam or using nanoparticles. They pair expensive PCMs with equally expensive enhancers (copper or aluminum foams, carbon nanotubes, graphene, etc.) to maximize performance, ignoring cost/benefit ratios.

There is a lack of studies optimizing the use of waste chips to enable the economic viability of high-grade n-Octadecane systems. The innovative value of this paper results from addressing the “Sustainability Paradox” of modern thermal storage materials (where energy storage is made using materials with high embodied carbon). It is validated as a pathway to reduce the cost of heat storage by using a waste byproduct (aluminum chips) that has a very low embodied carbon combined with high-performance n-octadecane, feasible for bulk applications (e.g., building envelopes, battery thermal management, and solar tanks) where it was previously too expensive. Unlike nanoparticles that pose health risks and tend to clump, aluminum chips are macro-scale, safe to handle, and can form a percolation network more easily at lower volume fractions if the chip geometry is optimized.

A comparative analysis of the cost, environmental friendliness, and difficulty of preparation, presented in Table 1, reveals a distinct advantage for recycled aluminum chips, especially for industrial-scale applications.

In that context, in this experimental study, a new PCM composite based on n-octadecane and aluminum chips was developed and analyzed. N-octadecane was selected as the PCM due to its melting temperature, which is within the range required for thermal comfort in buildings. Aluminum chips resulting from industrial waste were proposed as thermal enhancers, but also to promote and sustain the materials’ circularity. These wastes are abundant and cheap and can represent a valuable resource in the production of new composites.

Thermal parameters (thermal conductivity, latent heat, and diffusivity) and the transition range were analyzed as essential parameters in optimizing the composition of PCMs. Then, a correlation of composite thermal parameters with climate conditions was performed to determine the volume of material required to absorb the solar heat gain.

## 2. Materials and Methods

**Figure 1:**
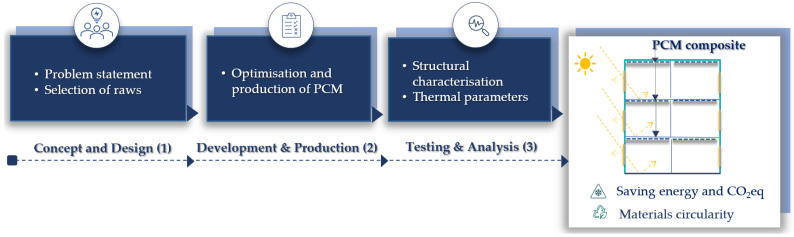
Product design process.

Raw materials and composites were produced and tested in the laboratory. The product design process was settled in a few stages (Concept and Design (1), Development and Production (2), Testing and Analysis (3)). Figure 1 highlights the potential of using the PCM composites in creating a passive means to a healthy internal environment.

Aluminum chips of different sizes and lengths were generated during the manufacturing of doors and windows, mostly obtained by drilling. An important advantage of using these chips is the simple process of reusing. Most workshops that use aluminum profiles for double-glazed windows do not use lubrication either in the sawing or the drilling processes. The aluminum chips were acquired from a local workshop. Their value is low; the recycling firms pay for the chips under 4 USD/Kg. The ratio of aluminum chips embedded in the PCM composite was optimized for maximum improvement in the thermal parameter, as well as to avoid the sinking of bigger particles during the melting process.

### 2.1. Raw Materials

The n-octadecane is part of the paraffin family, having a chemical formula (CnH2n + 2) where the melting temperature depends on the carbon chain length. The compounds tht have n between 5–15 are liquids at room temperature, and those with n ≥ 16 are solids. For the production of composite materials, n-octadecane (C_18_H_38_), with a melting point in the range of 28–30 °C and a heat capacity of 2.22 J/K·g, was selected. The thermophysical properties of n-octadecane (C_18_H_38_) are presented in Table 2 [31]. The thermal conductivity of n-octadecane during the transition phase varied from 0.198 W/m·K (solid) to 0.222 W/m·K (liquid).

The chemical composition and thermophysical properties of aluminum are presented in Table 3 and Table 4. The result of the chemical analysis shows that Al chips contain 0.25% Si, 0.23% Fe, and 0.35–0.6% Mg, and less than 0.15% Ti. The chemical content is in the range of the primary aluminum composition.

Aluminum chips were sieved and separated according to their shape factor so as to give a highly porous self-supporting structure that was made of high-shape-factor chips. Most chips that resulted from the drilling process possessed a high shape factor. Using sieved particles, two fractions (long, with a shape factor of over 40, and short, with a shape factor of around 15) were used in the case of aluminum alloy, with apparent densities of 0.7 g/cm^3^ and 0.14 g/cm^3^.

The resulting composites (n-octadecane-Al-long and n-octadecane-Al-short) were tested in the laboratory, and their thermophysical properties were analyzed.

**Figure 2:**
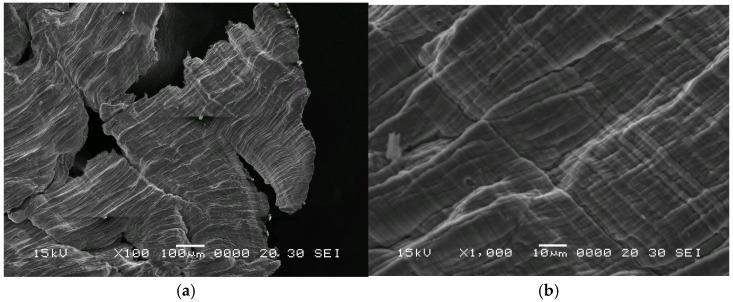
SEM analysis of the 6060 series aluminum chips: low magnification (**a**) and high magnification (**b**).

The microstructure of the aluminum chips was analyzed using scanning electron microscopy (SEM) (Jeol 5600-LV microscope—Akishima, Tokyo, Japan), Figure 2. The SEM images show that Al chips present a high aspect ratio and cracks, which are typical of machining chips.

### 2.2. Preparation of PCM Composites

Recycled aluminum chips were added in varying percentages by volume to manufacture the PCM composites. (Table 5). The metal content (% vol.) that was embedded in the PCM composites was determined from conditions that maintained a high energy absorption capacity. The resulting minimal quantity was, at 7%, for the high shape factor chips n-octadecane-Al-long, and 7.5% for the lower shape factor chips n-octadecane-Al-short.

The appropriate weight of the aluminum chips was measured on a laboratory balance and placed in a polyethylene mold. The low apparent density of the chips describes the high porosity between the chips that allows the melted octadecane (with a temperature of 40 °C) to easily infiltrate them. No degassing or special cleaning was needed. To manufacture cylindrical samples for thermal conductivity measurements by eliminating the volumetric shrinkage during the solidification process, an increased volume of n-octadecane was used, and after solidification, the excess was cut off. No significant porosity was observed inside the composites.

### 2.3. Experimental Study and Methods

Differential thermal analyses (DTA) were carried out in air at a constant heating rate of 10 °C min^−1^ on a modified MOM Derivatograph (MOM, Budapest, Hungary). For the isothermal measurements, the samples were heated to the desired temperatures at 10 °C min^−1^ and then maintained at a constant temperature until the melting was completed.

The thermal conductivity of the composites was established using a steady-state heat method. Temperatures were measured with chromel–alumel thermocouples. The thermal conductivity was measured on a ‘homemade’ setup in a temperature range of 25–35 °C. For each measurement, the steady-state unidirectional heat flow was achieved and maintained for at least 30 min prior to the measurements. The heat loss during the experiment was minimized during the measurements since the setup was isolated with 10 cm basalt wool. The experimental uncertainty is around 5–7%. Every measurement was repeated 3 times, and the presented value is their mean value. The other thermophysical parameters (specific heat (Cp), thermal diffusivity (α), specific heat capacity, and density (ρ)) were then calculated [20].

SEM images were taken on a Jeol 5600-LV microscope (Akishima, Tokyo, Japan) in a secondary electron configuration that was coupled with an energy-dispersive X-ray (EDX) spectrometer UltimMAX65 (Oxford Instruments, Aztec software, version 4.2, High Wycombe, UK).

The heat flux given by the total solar radiation (direct and diffuse) ΦI* (W) was determined based on C107/7-02, considering an average solar radiation of 172 W/m^2^ (Cluj-Napoca, July) [34,35].

## 3. Results and Discussion

### 3.1. Thermal Characterization of the PCM Composite

#### 3.1.1. Non-Isothermal DTA Analysis

**Figure 3:**
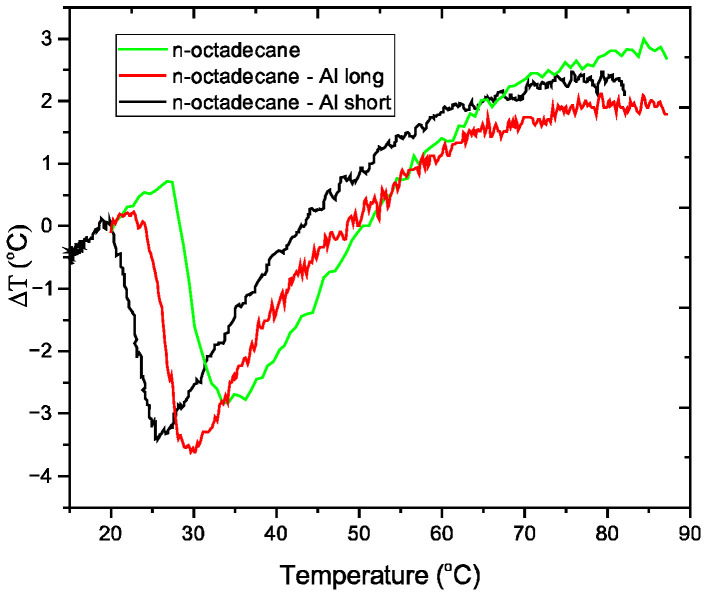
DTA curves for the samples studied.

Thermal analysis was performed on three sample types (pure n-octadecane, 7 vol.% n-octadecane-Al-long, and 7.5 vol.% n-octadecane-Al-short) using a modified MOM Hungary Derivatograph in air, with a constant heating rate of 10 °C/min. All samples exhibited a distinct endothermic peak corresponding to the melting of n-octadecane (Figure 3). The area of these peaks, which represents the total energy absorbed (latent heat), is proportional to the mass of n-octadecane, as the aluminum chips are thermally conductive but do not undergo a phase change in this range. While the aluminum does not melt, its addition strongly influences the melting process. A clear trend was observed: as the aluminum content increased, the melting peak temperature (T_p_) shifted to lower temperatures.

This phenomenon is attributed to an instrumental artifact known as thermal lag. PCMs, like n-octadecane, inherently combine high latent heat storage with low thermal conductivity. During a dynamic heating scan, this creates a significant internal thermal gradient; the heat cannot penetrate the bulk of the sample fast enough to keep it in equilibrium with the instrument’s programmed temperature. Consequently, the sample holder’s thermocouple (which measures the T_p_) is at a higher temperature than the average temperature of the melting PCM, artificially inflating the measured peak temperature.

The incorporation of highly conductive aluminum chips introduces efficient heat-transfer pathways throughout the composite. This enhancement in overall thermal diffusivity reduces internal temperature gradients and minimizes thermal lag. As the aluminum content increases up to 7.5%, the magnitude of this artifact diminishes progressively. The differential thermal analysis (DTA) peak thus appears to shift toward lower temperatures because the measured T_p_ more accurately represents the true thermodynamic melting point.

To quantify this thermal lag effect, the Kissinger equation was employed in a novel manner [36]. While typically used to find activation energy (E_a_) from scans at different heating rates (β), we instead used a fixed literature-based activation energy for octadecane (e.g., 8.78 kJ/mol [37]) to calculate an “equivalent heating rate” (β_eq_). This β_eq_ quantifies the effective heating rate that the sample experiences as its thermal lag reduced. The results in Table 6 clearly show that the sample with higher conductivity (7.5% Al) behaves as if it is being heated at a slower, more equitable rate.

#### 3.1.2. Isothermal Melting Analysis

**Figure 4:**
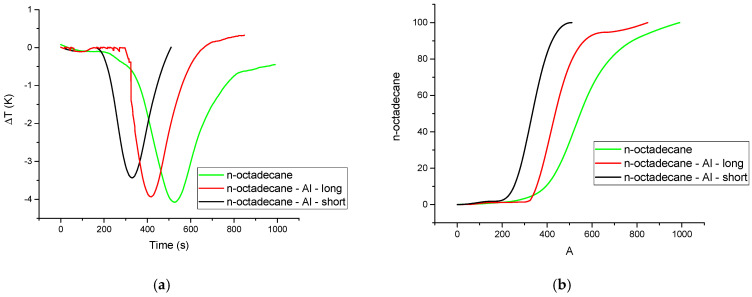
Isothermal melting curves (**a**) and melted fraction with time (**b**).

To confirm that the aluminum additives accelerate the melting process under practical, isothermal conditions (our primary goal), a separate isothermal analysis was performed. All isotherms exhibit a single endothermic peak, corresponding to the melting process at a constant temperature. The melting process begins after a distinct incubation time (τ), which is required for the system to equilibrate and for stable melting to commence. The transformed fraction (x) at any time (t) was determined by integrating the partial area of the peak [38]. The resulting plot of x vs. t yields the characteristic sigmoid curve that is common to phase-change kinetics, which starts slow, accelerates, and then slows as the final solid fraction is consumed (Figure 4).

As hypothesized, the presence of high-conductivity aluminum chips considerably accelerates the melting process. The sigmoid curves for the composites are significantly steeper, indicating a faster overall transformation rate. This acceleration is evident in the incubation time, which was determined graphically by extrapolating the main transformation curve back to the time axis. Compared to pure n-octadecane (τ = 392 s), the incubation time decreased by approximately 14% for the n-octadecane-Al-long sample (336 s) and by 36% for the n-octadecane-Al-short sample (251 s).

The full analysis in Table 2 further confirms that the aluminum–octadecane composites not only start melting earlier (lower τ) but also complete the entire phase transition in a fraction of the time required for pure octadecane, demonstrating the beneficial effect of thermal conductivity enhancement on phase-change kinetics.

#### 3.1.3. Thermophysical Parameters

The thermophysical parameters of PCM composites are presented in Table 7 and Table 8. Improvements in the thermal conductivity were obtained for all developed composite materials. An increase from 0.22 W/m·K (n-octadecane) to 1.54 W/m·K was obtained in the case of n-octadecane-Al-long. A lower improvement up to 0.44 W/m·K was recorded in the case of n-octadecane-Al-short.

In the composite with short chips, they are largely isolated from one another by a layer of octadecane. Heat must travel from one aluminum chip, across the octadecane gap, and into the next chip. The interface between the metal and the octadecane acts as a thermal bottleneck due to the low conductivity of the matrix material. The composite’s overall conductivity is only double the conductivity of pure octadecane.

In the case of the composite with long chips, they are much more effective at forming more continuous pathways. The long chips easily touch and overlap, creating a continuous, three-dimensional thermal percolation network that spans the entire composite structure. Particles with a high aspect ratio (like long chips) are far superior because they create a thermal short circuit through the composite.

The improvement in thermal parameters was highlighted by other researchers who embedded the expanded graphite, nanosilica, or other metals in the composition of PCM. Rawat & Joshi [39] proposed the use of polypyrrole and expanded graphite in equal percentages in the composition of octadecane and paraffin wax. The results show an increase of thermal conductivity from 0.17 W/m·K (octadecane) to 1.69 W/m·K (octadecane with a mixture of 20%), respectively, from 0.19 W/m·K (paraffin wax) up to 1.41 W/m·K (paraffin wax with a mixture of 15%). To increase the storage/release capacity of n-octadecane, the authors, Guo et al. [40], proposed the encapsulation of n-octadecane with silica in different ratios, showing an increase of about 100%, from 0.14 W/m·K (n-octadecane) up to 0.28W/m·K.

Ju et al. [41] integrated an n-octadecane (ODE)/SiO_2_ nanocapsule in the composition of cementitious materials as a partial cement replacement (0–10% wt). A reduction in the peak temperature of 3.9 °C was recorded for the maximum percentage of nanocapsules, and a reduction in compressive strength up to 19.5% (from 48 MPa to 38.7 MPa), compared to reference samples.

Recycled aluminum chips offer a significantly higher thermal conductivity enhancement amplitude per unit cost compared to nanosilica. Aluminum provides 1.75 times the conductivity increase (7× vs. 4×) while utilizing a near-zero cost waste material, whereas nanosilica is a specialized, synthesized material with a high cost per gram.

Aluminum chips were effective in improving the thermal conductivity of composites and their speed to store/release heat. The densities of the samples increased by up to 15%, from 0.80 g/cm^3^ (n-octadecane) to 0.92 g/cm^3^ (n-octadecane-Al-short). Effusivity and diffusivity, the other two thermal parameters, were improved for both types of composites (Table 8).

**Figure 5:**
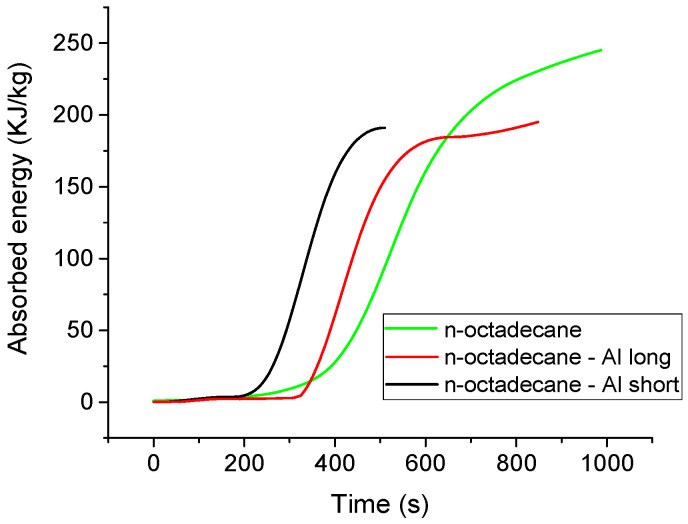
Absorbed energy versus time.

The most effective PCM composites seem to be n-octadecane-Al-long, whose effusivity values were increased by 166%, up to 1543 Ws^1/2^ m^−2^ K^−1^, respectively, with about 590%, which represents 9.95 × 10^−7^ m^2^/s in comparison with n-octadecane when diffusivity was considered. The energy absorption by the composites (Figure 5) was completed in 510 s for n-octadecane-Al-short, 850 s for n-octadecane-Al-long, and 990 s for pure n-octadecane.

Using recycled aluminum chips as thermal enhancers presents a compelling case, particularly when considering economics and environmental impact, with the following advantages:

- Cost efficiency: The material cost is exceptionally low, maximizing the cost/benefit ratio for thermal enhancement.
- Superior thermal enhancement: Due to the metallic nature and high intrinsic thermal conductivity of aluminum, it provides a much higher absolute conductivity increase compared to most oxide or ceramic fillers SiO_2_.
- Environmental sustainability: Recycling aluminum supports the circular economy model by reusing waste material, with almost no supplementary energy input.

The disadvantages of using these composites consist of processing and handling inconveniences: Loose chips have low-density, high-volume waste that can sink in the liquid PCM matrix, requiring specialized selection steps to ensure network formation and prevent sinking/segregation during phase-change cycles. Regarding the oxide layer impact, the native oxide layer on the surface of the chips, which insulates, can increase interfacial thermal resistance between the metal network and the octadecane, somewhat reducing the conductivity if the thermal contact becomes poor. Having long chips means a low number of contacts, and so the negative effect is somewhat reduced.

The relationship between increasing the aluminum chip content (thermal enhancer) and the resulting latent heat reduction (energy storage capacity) is a fundamental trade-off when considering a phase-change material composite design.

The primary objective of adding highly conductive metallic chips is to significantly boost thermal conductivity, thereby improving the charging/discharging rates and system power output. However, since the chips possess negligible latent heat, increasing their volume fraction directly and linearly reduces the system’s total latent heat storage capacity.

Optimization aims to maximize thermal conductivity while minimizing the required metallic chips. The simplest and most effective strategy is to utilize high-aspect-ratio chips. This chip morphology lowers the thermal percolation threshold, ensuring that a continuous, highly conductive network forms at a minimum volume of aluminum. This maximizes the remaining PCM volume, thus achieving the highest possible latent heat storage capacity for a given target thermal performance.

Utilizing these chips can be extended to all paraffin-based PCMs since they are inert to each other.

### 3.2. Mass of Composite Required for Passive Cooling

**Figure 6:**
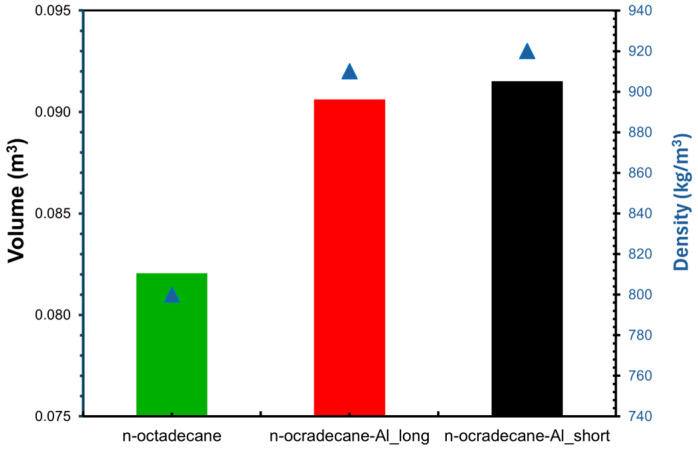
Volume of PCM composite required for cooling the room (the colored bar – volume and the composite’s density (blue triangle).

The quantity of PCM composite was dimensioned when considering the flux of solar radiation through glazing. For Cluj-Napoca, the average value of solar radiation I (W) in July is about 172 W/m^2^ day [34], therefore, resulting in an average heat flux ΦI* (W) value of 4.5 kWh/day. The geometrical characteristics of the room and construction elements are the following: the floor area (A_floor_) is 18 m^2,^ and the glazing area (A_g_) is 3.6 m^2^. This results in a required quantity of PCM of a maximum 84.2 kg for n-octadecane-Al-short and a minimum 82.5 kg for n-octadecane-Al-long compared to 65.6 n-octadecane. The PCM composites are considered to be placed in each room on the floor level under the slab. Therefore, the volume of each composite was determined while considering the maximum mass (kg) required to absorb the solar radiation and the floor area. It results in a volume of 0.083 m^3^ for n-octadecane, 0.092 m^3^ for n-octadecane-Al-short, and 0.091 for n-octadecane-Al-long (Figure 6); an increase of up to 11% is needed to absorb the solar heat gain for optimized PCM in comparison to n-octadecane.

**Figure 7:**
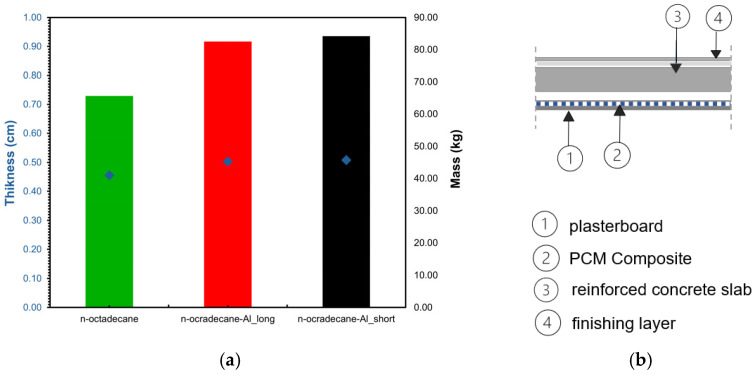
Thickness of the PCM composite calculated at the maximum mass required (the colored bar), the corresponding thickness (blue diamond) (**a**), and the construction scheme (**b**).

The maximum thickness of the layer for n-octadecane is 0.46 cm, and 0.51cm for the composite with aluminum chips (Figure 7), which allows for the energy cost savings that are required for the HVAC system to maintain thermal comfort in buildings. 

The PCM composites were sized to absorb the solar radiation on hot days, thus reducing the air conditioning operating times. Considering that the solar heat input is around 4.5 kWh/day, this results in a quantity of 150 kg CO_2_eq for a period of 90 days, which can be saved each year by using these phase-change composites. The conversion of electrical energy into equivalent CO_2_ emissions was done using the conversion factors given by the European Environment Agency [42]. Extrapolating this value for a longer period, about 15 years, which can be considered between two major renovations, results in a reduction of 2.3 t CO_2_eq. In fifteen years, this value depends on the type of energy used for cooling the buildings, as well as the climatic parameters.

These materials have a low degradation in time and resist a high number of absorbed/released heat cycles without any significant change in their properties. Firman et al. [43] demonstrated that after 10,000 cycles, the heat-absorbing capacity of paraffin-based PCMs remained over 80% of the initial value.

A future direction of this research is the optimization of chip alignment in the primary direction of heat flow to boost the required conductivity by using less material than the randomly oriented chips option. The thermal performance and energy savings of the resulting PCM composite will be evaluated by extending the laboratory tests to buildings in real environmental conditions.

## 4. Conclusions

In this experimental study, different types of PCM composites were analyzed and proposed for thermal control in dormitory rooms. These composites were made of n-octadecane and recycled aluminum chips for implementation as a thermal conductivity enhancer. PCM composites were optimized while considering maximum improvements in thermal parameters, as well as their durability.

The main findings of this experimental study are the following:

- Recycled aluminum was selected for composite production. This was considered a simple and environmentally friendly way to increase the materials’ circularity, as well as to improve the thermal parameters of the composites. Al chips were used in two different percentages and lengths to exploit the maximum of their potential. The results show an increase of thermal conductivity from 0.22 W/m·K (n-octadecane) to 1.54 W/m·K, by approximately 600%, in the case of n-octadecane-Al-long. This was more effective than n-octadecane-Al-short, since its length permits fewer contact points for heat transfer.
- Looking at the DTA curves, it was observed that the melting peak temperature (T_p_) shifted to lower temperatures as the aluminum content increased.
- The volume of PCM composites varied from 0.083 m^3^ for n-octadecane and 0.092 for n-octadecane-Al-short, which represents an increase of about 11%, which is required to absorb solar heat gains by the optimized PCM composite.

## Figures and Tables

**Table 1:** Comparison of cost, environmental friendliness, and preparation difficulty of some thermal enhancers for PCM [25,26,27,28,29,30].

Feature	Recycled Aluminum Chips	Nanosilica (SiO_2_)	Expanded Graphite (EG)/ Graphene
Preparation Difficulty	Low (Mechanical) Method: Cleaning, sieving, and simple physical dispersion. Challenge: Settling issues (manageable with packing factor).	High (Chemical) Method: Sol-gel synthesis or in-situ polymerization. Challenge: Agglomeration of nanoparticles is a major issue; it requires ultrasonic dispersion or surface functionalization.	High (Complex) Method: Thermal expansion and vacuum impregnation. Challenge: A highly porous structure makes uniform PCM infiltration difficult without a vacuum; volume expansion creates handling issues.
Cost Effectiveness	Very high: Sourced from industrial waste (machining scrap). Material Cost: Very low.	Moderate (industrial fumed silica) to low (specialized/functionalized nano). Issue: High loading is required for a significant thermal boost, increasing the total cost.	Low (natural graphite) to moderate to high (expanded graphite/graphene). Issue: High processing costs make it prohibitive for large-scale building/thermal battery applications.
Environmental Friendliness	Excellent for circular economy practices. Energy/Carbon Footprint: Very low. Waste: Reduce landfilling.	Moderate energy/carbon footprint: Production involves energy-intensive electric furnaces. Risk: Nanoparticles pose potential inhalation/toxicity risks during handling and disposal.	Moderate to poor energy/carbon footprint: Graphitization requires extreme temperatures. Risk: EG production often uses strong acids and oxidizers.

**Table 2:** Properties of n-octadecane [31].

PCM	Molar Mass (g/moL)	Density (g/cm^3^)	Melting Point (°C)	Viscosity (mPa·s) (20 °C)	Heat Capacity (J/(K·g))	Thermal Conductivity (W/m·K)
n-octadecane (C_18_H_38_)	254.494	0.800	28–30	4.21	2.222	0.222 (solid)

**Table 3:** Chemical composition of aluminum (%).

Alloy	Si	Fe	Cu	Mn	Mg	Zn	Ti	Al
Al 6060 [32]	0.3–0.6	0.1–0.3	<0.1	<0.1	0.35–0.6	<0.1	<0.15	R*
Al chips	0.25	0.23	0.05	0.05	0.35	0	0.05	R*

R*—remainder.

**Table 4:** Thermophysical properties of aluminum alloy.

Alloy	Density (Kg/m^3^)	Specific Heat Capacity (J/kg·K)	Thermal Conductivity (W/m·K)
Al 6060 [33]	2700 (at 20 °C)	898	200–220

**Table 5:** Properties of PCM composites.

Sample	Metal Content (Vol.%)
n-octadecane	0
n-octadecane-Al long	7
n-octadecane-Al short	7.5

**Table 6:** Equivalent heating rates.

T_peak_ (°C)	Al Content wt.%	Heating Rates (K/min)
34.8	0 (n-octadecane)	9.97
30	7 (n-octadecane-Al-long)	9.29
26.0	7.5 (n-octadecane-Al-short)	8.75

**Table 7:** Properties of PCM composite.

Sample	Density (g/cm^3^)	Thermal Conductivity (W/m·K)	Latent Heat of Fusion (kJ/kg)	Specific Heat Capacity (J/kg·K)
n-octadecane	0.80	0.22	245	1908
n-octadecane-Al-short	0.92	0.44	191	1690
n-octadecane-Al-long	0.91	1.54	195	1700

**Table 8:** PCM composites.

Sample	Effusivity (Ws^1/2^ m^−2^ K^−1^)	Percentage of Increase (%)	Diffusivity (m^2^/s)	Percentage of Increase (%)
n-octadecane	579	-	1.44 × 10^−7^	-
n-octadecane Al-short	827	43	2.83 × 10^−7^	96
n-octadecane Al-long	1543	166	9.95 × 10^−7^	590

## Data Availability

The original contributions presented in this study are included in the article. Further inquiries can be directed to the corresponding author.
